# A novel model for predicting posthepatectomy liver failure in patients with hepatocellular carcinoma

**DOI:** 10.1371/journal.pone.0219219

**Published:** 2019-07-03

**Authors:** Wei Peng, Jia-Wu Li, Xiao-Yun Zhang, Chuan Li, Tian-Fu Wen, Lv-Nan Yan, Jia-Yin Yang

**Affiliations:** 1 Department of Liver Surgery & Liver Transplantation Center, West China Hospital, Sichuan University, Chengdu, Sichuan Province, China; 2 Department of Ultrasound, West China Hospital, Sichuan University, Chengdu, Sichuan Province, China; China Medical University Hospital, TAIWAN

## Abstract

Posthepatectomy liver failure (PHLF) is the most leading cause of mortality following hepatectomy in patients with hepatocellular carcinoma (HCC). Platelet count was reported to be a simple but useful indicator of liver cirrhosis and function of spleen. Spleen stiffness (SS) was used to evaluate the morphological change of spleen and was reported to be related to liver cirrhosis and portal hypertension. However, the predictive value of platelet to spleen stiffness ratio (PSR) on PHLF remains unknown. A retrospective study was performed to analyze 158 patients with HCC following hepatectomy from August 2015 to February 2016. Univariate and multivariate analyses were performed to evaluate the value of each risk factor for predicting PHLF. The predictive efficiency of the risk factors was evaluated by receiver operating characteristic (ROC) curve. PHLF occured in 23 (14.6%) patients. PSR (P<0.001, odds ratio (OR) = 0.622, 95% confidence interval (CI) 0.493~0.784), hepatic inflow occlusion (HIO) (P = 0.003, OR = 1.044, 95% CI 1.015~1.075) and major hepatectomy (P = 0.019, OR = 5.967, 95% CI 1.346~26.443) were demonstrated to be the independent predictive factors for development of PHLF in a multivariate analysis. Results of the present study suggested PSR is a novel and non-invasive model for predicting PHLF in patients with HCC.

## Introduction

Hepatocellular carcinoma (HCC) is one of the most common malignancies and is the third leading cause of cancer related deaths worldwide[1]. The predominant risk factor for HCC is chronic infection of hepatitis B virus (HBV) in China[2]. More than half of the new cases and deaths occurred in China because of the high prevalence of HBV[3]. Hepatectomy is widely accepted as a curative treatment for HCC patients even though liver transplantation is the ideal treatment[4]. The reported incidence of posthepatectomy liver failure (PHLF) is as high as 12% according to the definition by International Study Group of Liver Surgery (ISGLS)[5]. Therefore, continuous effort to identify risk factors for development of PHLF remains necessary.

A majority of patients diagnosed with HCC were accompanied with underlying liver fibrosis or cirrhosis. Distortion of the normal liver architecture and formation fibrous septum in patients with liver cirrhosis would increase the resistance to portal vein flow, resulting in portal hypertension, hypersplenism, and resultant thrombocytopenia[6, 7]. Platelet count was reported to be a simple but useful indicator of liver cirrhosis and function of spleen[8]. Furthermore, recent studies found platelet a predictor for PHLF[9, 10]. Spleen stiffness (SS), measured by ultrasound-based technologies, was used to evaluate the morphological change of spleen and was reported to be related to liver cirrhosis and portal hypertension[11, 12]. Theoretically, in patients with liver cirrhosis, spleen tissues showed passive congestion with a dilated sinus, diffuse α-smooth muscle actin expression of sinusoidal mesenchymal cells, and deposition of collagen fibers on the perisinusoidal wall[13]. Therefore, increased SS was reported to be an indirect indicator for liver cirrhosis and portal hypertension.

However, the clinical value of platelet to spleen stiffness ratio (PSR) which represents the integration of functional and morphological change of spleen remains unknown. The present study was designed to evaluate the predictive value of PSR on development of PHLF in patients with HCC.

## Patients and methods

This is a retrospective analysis of prospectively collected data of newly diagnosed HCC patients following hepatectomy at our department from August 2015 to February 2016. The laboratory and radiological data of each patient were retrieved from the prospectively maintained database. To protect patients’ privacy, authors had no access to information that could identify individual participants during or after data collection. We excluded the patients who (1) received preoperative antitumor treatment; (2) previous or present splenectomy or splenic embolization. HCC and liver cirrhosis were confirmed by a postoperative pathological examination. The study was approved by the Ethics Committee of West China Hospital, Sichuan University. All methods were performed in accordance with the relevant guidelines and regulations.

### Measurement of SS

SS value was obtained by elastography point quantification examination in the morning after at least 8 hours of fasting after a complete abdominal ultrasonic examination. The measurement was performed by one experienced physician with an iU22 ultrasonic system (Philips iU22, Philips Medical Systems, Royal Philips Electronics, Netherlands). SS measurement was performed in vessel-free area of the background spleen during a 5 seconds breath hold at inspiration with the probe positioned in an intercostal space where the spleen was correctly visualized by ultrasonic system[14]. For each patient, SS value was accepted only if the success rate (ratio of the number of successful measurements to the total number of acquisitions) was more than 60% and the interquartile range was less than 30% of the median value[15]. The mean value of 10 successful measurements was calculated and expressed in kilopascals (kPa).

### Intraoperative care

All surgeries were performed by experienced surgeons with intraoperative ultrasonographic guidance. Intermittent hepatic inflow occlusion (HIO) and low central venous pressure anesthesia was applied to control bleeding in most cases[16]. Majority of liver dissections were carried out by ultrasonic dissector and clamp crushing techniques, water jet was an alternative choice in some cases[17]. The range of hepatectomy was determined by the extent of tumor progression and liver functional reserve. Major hepatectomy was defined as resection of 3 Couinaud’s segments or more.

### Definitions of PHLF

PHLF was defined as a postoperatively acquired deterioration in the ability of the liver to maintain its synthetic, excretory, and detoxifying functions, which are characterized by an increased INR and concomitant hyperbilirubinemia (INR>1.14 and serum bilirubin more than 28 μmol/L according to the normal limits of our hospital) on or after postoperative day 5 according to the ISGLS definition[18].

### Statistical analysis

Categorical data were expressed as numbers (percentage) and compared by the chi-square test or Fisher exact test. Continuous variables were expressed as mean (range) and compared by the independent sample t test. Candidate predictors of PHLF considered in this analysis were determined by a univariate analysis. Logistic regression analysis was used to evaluate the risk factors for PHLF. Receiver operating characteristic (ROC) curves were calculated for each of the predictive factors for PHLF and each area under the curve (AUC) were computed. P values less than 0.05 were considered statistically significant. All statistical analyses were performed with SPSS software version 21.0 (SPSS Company, Chicago, IL, United States).

## Results

### Main clinical features

From August 2015 to February 2016, 164 patients were enrolled based on the inclusion criteria and 6 of them were excluded from the study: 3 had hepatectomy and splenectomy at the same time, 3 had previous splenectomy. A total of 158 patients were finally analyzed based on the inclusion and exclusion criteria. Among them, 129 (81.6%) were male and 29 (18.4%) were female. The main clinical features of the whole patient population were described in Table 1.

**Table 1:** Main clinical features of patient population according to development of PHLF.

Factors	No PHLF n = 135 (85.4%)	PHLF n = 23 (14.6%)	P value
Age (years)	52.6±11.9	50.8±10.8	0.509
Gender (Female/Male)	24/111	5/18	0.770
Etiology (HBV/Others)	105/30	16/7	0.427
Total bilirubin (μmol/L)	15.39±5.60	13.86±6.06	0.234
ALT (IU/L)	41.84±23.19	36.91±15.71	0.206
AST (IU/L)	41.72±26.13	43.96±24.88	0.703
Albumin (g/L)	42.62±3.87	41.64±5.02	0.382
INR	1.03±0.10	1.03±0.09	0.943
Platelet count (<150*10^9/L VS ≥150*10^9/L)	72/63	17/6	0.073
Tumor diameter (cm)	5.67±3.49	7.76±3.99	0.010
Cirrhosis (Yes/No)	59/76	19/4	0.001
ICG-R15 (%)	4.51±3.92	5.52±3.42	0.317
Spleen stiffness (kPa)	15.11±8.82	26.79±7.98	<0.001
PSR	12.73±8.82	4.42±3.05	<0.001
Operation duration (Min)	205.29±59.81	264.34±70.89	<0.001
HIO (Min)	26.79±18.42	41.78±27.30	0.001
Major hepatectomy (Yes/No)	66/69	19/4	0.003
Transfusion (Yes/No)	3/132	4/19	0.009

ALT = alanine aminotransferase; AST = Aspartate aminotransferase; INR = international normalized ratio; ICG-R15 = indocyanine green retention rate at 15 minutes; PSR = platelet to spleen stiffness ratio; HIO = hepatic inflow occlusion; PHLF = posthepatectomy liver failure.

### Univariate and multivariate analyses for PHLF

**Fig 1:**
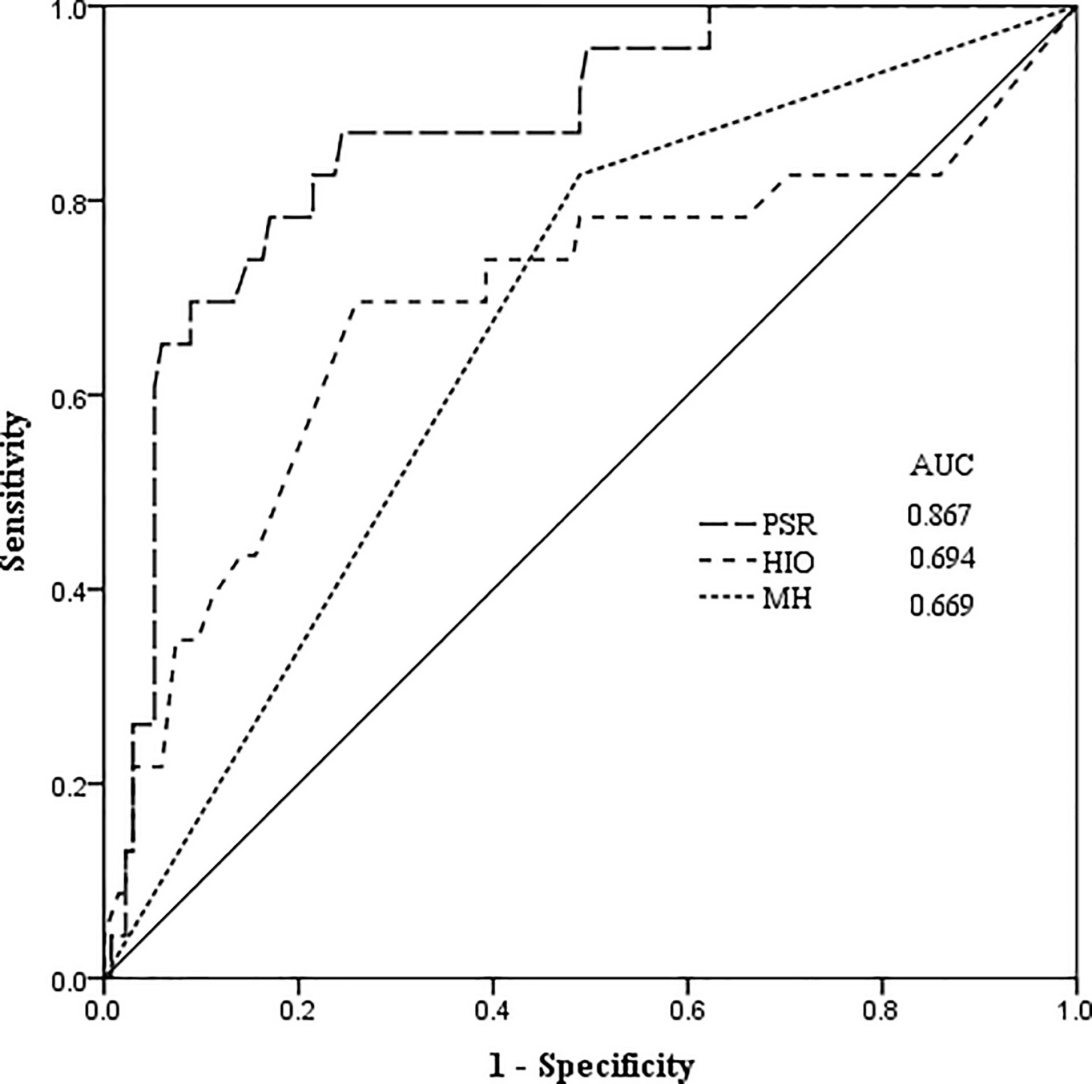
Predictive efficacy of PSR, HIO and MH for predicting PHLF. PSR = platelet to spleen stiffness ratio, HIO = hepatic inflow occlusion, MH = major hepatectomy, AUC = area under the curve.

PHLF developed in 23 (14.6%) patients according to the ISGLS definition. Univariate analysis suggested that tumor diameter, liver cirrhosis, SS, PSR, operation duration, HIO, major hepatectomy and intraoperative transfusion were significantly related to development of PHLF (Table 1). Multivariate analysis demonstrated that PSR (P<0.001, odds ratio (OR) = 0.622, 95% confidence interval (CI) 0.493~0.784), HIO (P = 0.003, OR = 1.044, 95% CI 1.015~1.075) and major hepatectomy (P = 0.019, OR = 5.967, 95% CI 1.346~26.443) were the independent predictive factors for development of PHLF in patients with HCC (Table 2). The area under ROC curve (AUC) of PSR (AUC 0.867, 95% CI 0.790~0.943) for predicting PHLF was greater than that of HIO (AUC 0.694, 95% CI 0.555~0.832) and major hepatectomy (AUC 0.669, 95% CI 0.559~0.778) (Fig 1). The optimal cut-off value of HIO was set at 40 min for predicting PHLF with a maximum joint sensitivity and specificity (sensitivity = 0.696, specificity = 0.741) while the optimal cut-off value of PSR was set at 4.60 for predicting PHLF (sensitivity = 0.696, specificity = 0.896).

**Table 2:** Predictive factors for PHLF in multivariate analysis.

Factors	P value	Odds ratio	95%CI
Age	0.762		
Gender (Male/Female)	0.302		
Total bilirubin	0.644		
ALT	0.249		
AST	0.174		
Albumin	0.392		
INR	0.596		
ICG-R15	0.623		
PLT(<150*10^9/L VS ≥150*10^9/L)	0.915		
Liver cirrhosis (Yes VS No)	0.767		
Tumor diameter	0.460		
Spleen stiffness	0.972		
PSR	<0.001	0.622	0.493~0.784
Operation duration	0.097		
HIO	0.003	1.044	1.015~1.075
Major hepatectomy (Yes VS No)	0.019	5.967	1.346~26.443
Transfusion (Yes VS No)	0.383		

PHLF = posthepatectomy liver failure; ALT = alanine aminotransferase; AST = Aspartate aminotransferase; INR = international normalized ratio; ICG-R15 = indocyanine green retention rate at 15 minutes; PSR = platelet to spleen stiffness ratio; HIO = hepatic inflow occlusion; CI = confidence interval.

### The predictive value of PSR on liver cirrhosis

**Fig 2:**
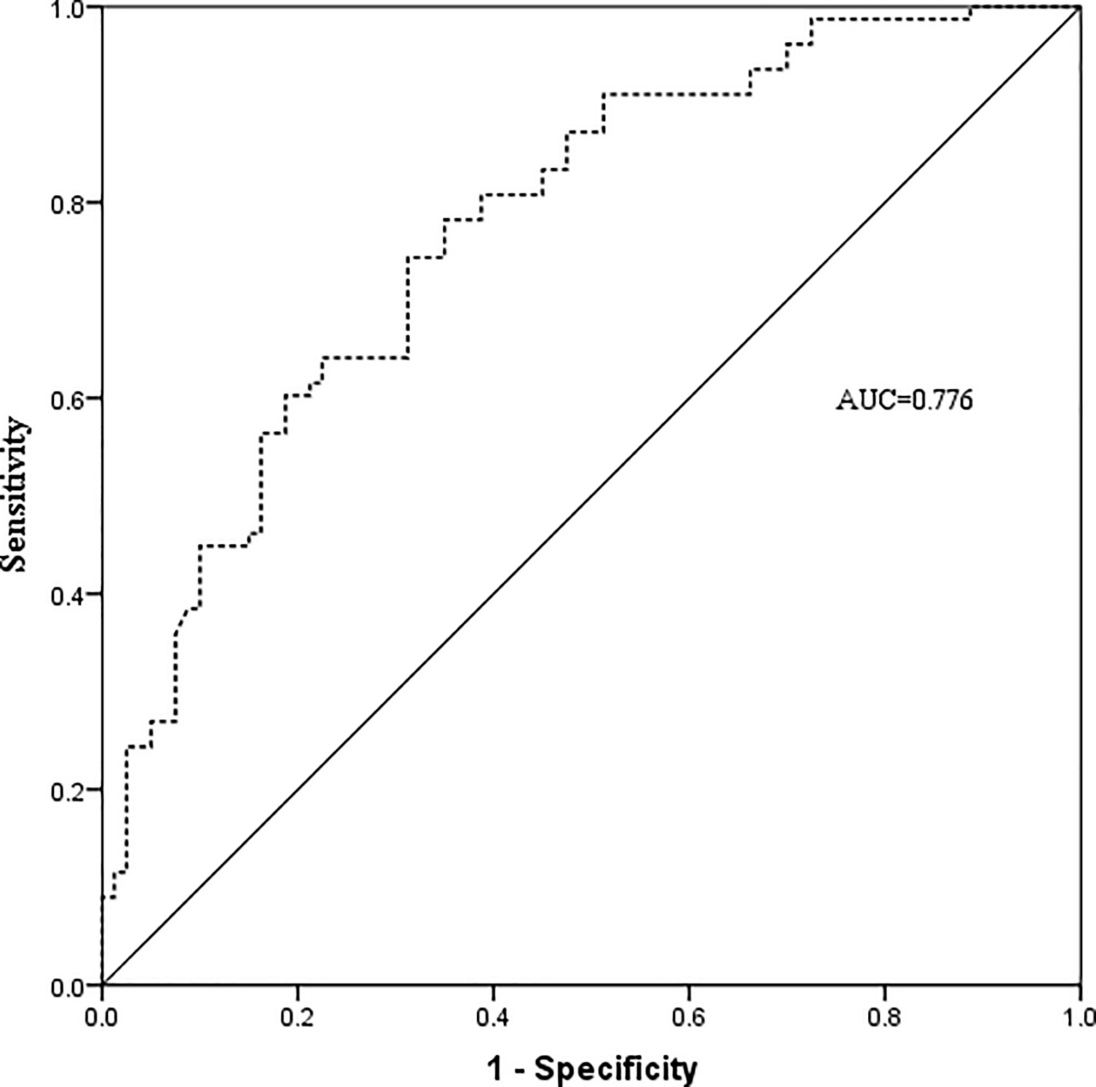
Predictive efficacy of PSR for predicting liver cirrhosis. PSR = platelet to spleen stiffness ratio, AUC = area under the curve.

Liver cirrhosis was diagnosed in 78 (49.4%) patients by a postoperative pathological examination. To clarify the predictive value of PSR on liver cirrhosis, a ROC analysis was performed (Fig 2). The AUC of PSR for predicting liver cirrhosis was 0.776 (P<0.001, 95%CI 0.704–0.847), and the optimal cut-off value was set at 10.0 for predicting liver cirrhosis with a maximum joint sensitivity and specificity (sensitivity = 0.782, specificity = 0.650).

## Discussion

PHLF is the most leading cause of mortality following hepatectomy, which has been studied by many researchers from different prospects. Most studies focused on liver itself while little attention was paid to spleen[19, 20]. In the present study, for the first time, a novel and non-invasive model which was also an integration of splenic function and morphology was found to be an independent predictor for PHLF.

Platelet count, an indirect indicator of liver cirrhosis and hypersplenism was reported to be a predictor of both short-term and long-term outcomes of HCC following hepatectomy[10, 21]. Particularly, various studies found that a low preoperative platelet count (<150*10^9/L) was significantly related to development of PHLF[22, 23]. In the present study, patients developed PHLF had a higher incidence of low preoperative platelet (73.9%) than those who didn’t (53.3%), but the difference was not statistically significant (P = 0.073). Clinical evidence showed that a certain number of platelets were required for liver function recovery[9, 24]. Studies in mice revealed the potential mechanism for the effect of platelet on liver regeneration. Lesurtel M et al found that platelet-derived serotonin mediated the initiation of liver regeneration and mice with thrombocytopenia would fail to initiate cellular proliferation in liver[25]. Furthermore, platelets could also exert positive effects on liver regeneration through cooperation with liver sinusoidal endothelial cells, Kupffer cells and hepatocytes [26–28].

SS was reported to be a useful non-invasive assessment for liver cirrhosis and portal hypertension in recent years [14, 29]. But the effect of SS on PHLF is controversial. Wu et al found SS obtained by transient elastography was not a significant predictor for PHLF in patients with HCC [30]. In their study, the patients developed PHLF had a higher value of SS (48.0kPa) than those who didn’t (26.3kPa), but the difference was not statistically significant (P = 0.36). However, Marasco et al found patients developed PHLF had a higher value of SS (60.0kPa) than those who didn’t (35.0kPa) (P<0.05)[31]. In the present study, our results were in line with previous clinical studies, SS was found to be related to development of PHLF in univariate analysis but not confirmed to be an independent predictor in multivariate analysis.

To clarify the other potential mechanism of the effect of PSR on PHLF, we found PSR to be a predictor (P<0.001, AUC 0.776) for liver cirrhosis which was the predominant risk factor for PHLF. A low PSR means a relatively low platelet count and a high value of SS, low platelet count and increased SS are the indirect indicators for liver cirrhosis and portal hypertension. An overperfusion from portal vein to a cirrhotic liver might lead to hepatic sinusoidal endothelium injury, delayed liver function recovery and PHLF[32].

Prolonged HIO and major hepatectomy were found to be independent risk factors for PHLF by multivariate analysis in the present study. Major hepatectomy was a well-established risk factor for PHLF[33]. The effect of intermittent HIO on PHLF is controversial. Hester et al found prolonged HIO was related to increased risk of PHLF[34]. While Andreatos et al found prolonged HIO was not a risk factor for PHLF[35]. In our opinion, prolonged HIO and major hepatectomy were related to complicated procedures, massive bleeding and transfusion[36, 37], which might lead to PHLF.

A limitation of the present study is its retrospective design. We were not able to get the other parameters such as splenic venous flow and portal venous pressure gradient. Secondly, our statistical analysis was limited by a small number of cases, it would be more convincing if the new model PSR was validated in another validation set and compared with other established models. Future prospective study will be designed to explore the clinical value of spleen on liver regeneration and PHLF, and the underlying mechanisms.

## Conclusion

PSR, an integration of functional and morphological change of spleen, is a novel and non-invasive model for predicting PHLF in patients with HCC.

## Supporting information

S1 File STROBE statement. (DOCX)

## References

[1] Bray F, Ferlay J, Soerjomataram I, Siegel RL, Torre LA, Jemal A. Global cancer statistics 2018: GLOBOCAN estimates of incidence and mortality worldwide for 36 cancers in 185 countries. CA Cancer J Clin. 2018;68(6):394–424. 10.3322/caac.21492 30207593

[2] Chen W, Zheng R, Baade PD, Zhang S, Zeng H, Bray F, et al Cancer statistics in China, 2015. CA Cancer J Clin. 2016;66(2):115–32. 10.3322/caac.21338 26808342

[3] Polaris Observatory C. Global prevalence, treatment, and prevention of hepatitis B virus infection in 2016: a modelling study. Lancet Gastroenterol Hepatol. 2018;3(6):383–403. 10.1016/S2468-1253(18)30056-6 29599078

[4] Poon RT, Fan ST, Lo CM, Liu CL, Wong J. Long-term survival and pattern of recurrence after resection of small hepatocellular carcinoma in patients with preserved liver function: implications for a strategy of salvage transplantation. Annals of surgery. 2002;235(3):373–82. 10.1097/00000658-200203000-00009 11882759 PMC1422443

[5] Rahbari NN, Reissfelder C, Koch M, Elbers H, Striebel F, Buchler MW, et al The predictive value of postoperative clinical risk scores for outcome after hepatic resection: a validation analysis in 807 patients. Ann Surg Oncol. 2011;18(13):3640–9. 10.1245/s10434-011-1829-6 21674269

[6] D'Amico G, Garcia-Tsao G, Pagliaro L. Natural history and prognostic indicators of survival in cirrhosis: a systematic review of 118 studies. J Hepatol. 2006;44(1):217–31. 10.1016/j.jhep.2005.10.013 16298014

[7] Aryal B, Yamakuchi M, Shimizu T, Kadono J, Furoi A, Gejima K, et al Deciphering Platelet Kinetics in Diagnostic and Prognostic Evaluation of Hepatocellular Carcinoma. Can J Gastroenterol Hepatol. 2018;2018:9142672 10.1155/2018/9142672 30050894 PMC6040256

[8] Kurokawa T, Ohkohchi N. Platelets in liver disease, cancer and regeneration. World J Gastroenterol. 2017;23(18):3228–39. 10.3748/wjg.v23.i18.3228 28566882 PMC5434428

[9] Alkozai EM, Nijsten MW, de Jong KP, de Boer MT, Peeters PM, Slooff MJ, et al Immediate postoperative low platelet count is associated with delayed liver function recovery after partial liver resection. Annals of surgery. 2010;251(2):300–6. 10.1097/SLA.0b013e3181b76557 19779326

[10] Mehrabi A, Golriz M, Khajeh E, Ghamarnejad O, Probst P, Fonouni H, et al Meta-analysis of the prognostic role of perioperative platelet count in posthepatectomy liver failure and mortality. Br J Surg. 2018;105(10):1254–61. 10.1002/bjs.10906 29999190

[11] Takuma Y, Nouso K, Morimoto Y, Tomokuni J, Sahara A, Takabatake H, et al Portal Hypertension in Patients with Liver Cirrhosis: Diagnostic Accuracy of Spleen Stiffness. Radiology. 2016;279(2):609–19. 10.1148/radiol.2015150690 26588019

[12] Buechter M, Manka P, Theysohn JM, Reinboldt M, Canbay A, Kahraman A. Spleen stiffness is positively correlated with HVPG and decreases significantly after TIPS implantation. Dig Liver Dis. 2018;50(1):54–60. 10.1016/j.dld.2017.09.138 29102174

[13] Kondo R, Kage M, Iijima H, Fujimoto J, Nishimura T, Aizawa N, et al Pathological findings that contribute to tissue stiffness in the spleen of liver cirrhosis patients. Hepatol Res. 2018;48(12):1000–7. 10.1111/hepr.13195 29766631

[14] Colecchia A, Montrone L, Scaioli E, Bacchi-Reggiani ML, Colli A, Casazza G, et al Measurement of spleen stiffness to evaluate portal hypertension and the presence of esophageal varices in patients with HCV-related cirrhosis. Gastroenterology. 2012;143(3):646–54. 10.1053/j.gastro.2012.05.035 22643348

[15] Castera L, Forns X, Alberti A. Non-invasive evaluation of liver fibrosis using transient elastography. J Hepatol. 2008;48(5):835–47. 10.1016/j.jhep.2008.02.008 18334275

[16] Man K, Fan ST, Ng IO, Lo CM, Liu CL, Wong J. Prospective evaluation of Pringle maneuver in hepatectomy for liver tumors by a randomized study. Annals of surgery. 1997;226(6):704–11; discussion 11–3. 10.1097/00000658-199712000-00007 9409569 PMC1191142

[17] Poon RT. Current techniques of liver transection. HPB (Oxford). 2007;9(3):166–73. 18333217 10.1080/13651820701216182 PMC2063596

[18] Rahbari NN, Garden OJ, Padbury R, Brooke-Smith M, Crawford M, Adam R, et al Posthepatectomy liver failure: a definition and grading by the International Study Group of Liver Surgery (ISGLS). Surgery. 2011;149(5):713–24. 10.1016/j.surg.2010.10.001 21236455

[19] Shirata C, Kokudo T, Arita J, Akamatsu N, Kaneko J, Sakamoto Y, et al Albumin-Indocyanine Green Evaluation (ALICE) grade combined with portal hypertension to predict posthepatectomy liver failure. Hepatol Res. 2019. 10.1111/hepr.13327 30849786

[20] Nishio T, Taura K, Koyama Y, Tanabe K, Yamamoto G, Okuda Y, et al Prediction of posthepatectomy liver failure based on liver stiffness measurement in patients with hepatocellular carcinoma. Surgery. 2016;159(2):399–408. 10.1016/j.surg.2015.06.024 26209567

[21] Zhang Z, Zhang Y, Wang W, Hua Y, Liu L, Shen S, et al Thrombocytopenia and the outcomes of hepatectomy for hepatocellular carcinoma: a meta-analysis. J Surg Res. 2017;210:99–107. 10.1016/j.jss.2016.11.002 28457347

[22] Golriz M, Ghamarnejad O, Khajeh E, Sabagh M, Mieth M, Hoffmann K, et al Preoperative Thrombocytopenia May Predict Poor Surgical Outcome after Extended Hepatectomy. Can J Gastroenterol Hepatol. 2018;2018:1275720 10.1155/2018/1275720 30515369 PMC6236772

[23] Maithel SK, Kneuertz PJ, Kooby DA, Scoggins CR, Weber SM, Martin RC 2nd, et al Importance of low preoperative platelet count in selecting patients for resection of hepatocellular carcinoma: a multi-institutional analysis. J Am Coll Surg. 2011;212(4):638–48; discussion 48–50. 10.1016/j.jamcollsurg.2011.01.004 21463803 PMC3487706

[24] Wang HQ, Yang J, Yang JY, Wang WT, Yan LN. Low immediate postoperative platelet count is associated with hepatic insufficiency after hepatectomy. World J Gastroenterol. 2014;20(33):11871–7. 10.3748/wjg.v20.i33.11871 25206294 PMC4155380

[25] Lesurtel M, Graf R, Aleil B, Walther DJ, Tian Y, Jochum W, et al Platelet-derived serotonin mediates liver regeneration. Science. 2006;312(5770):104–7. 10.1126/science.1123842 16601191

[26] Kawasaki T, Murata S, Takahashi K, Nozaki R, Ohshiro Y, Ikeda N, et al Activation of human liver sinusoidal endothelial cell by human platelets induces hepatocyte proliferation. J Hepatol. 2010;53(4):648–54. 10.1016/j.jhep.2010.04.021 20615569

[27] Abshagen K, Eipel C, Kalff JC, Menger MD, Vollmar B. Loss of NF-kappaB activation in Kupffer cell-depleted mice impairs liver regeneration after partial hepatectomy. Am J Physiol Gastrointest Liver Physiol. 2007;292(6):G1570–7. 10.1152/ajpgi.00399.2006 17322066

[28] Murata S, Ohkohchi N, Matsuo R, Ikeda O, Myronovych A, Hoshi R. Platelets promote liver regeneration in early period after hepatectomy in mice. World journal of surgery. 2007;31(4):808–16. 10.1007/s00268-006-0772-3 17354025

[29] Zhu YL, Ding H, Fu TT, Peng SY, Chen SY, Luo JJ, et al Portal hypertension in hepatitis B-related cirrhosis: Diagnostic accuracy of liver and spleen stiffness by 2-D shear-wave elastography. Hepatol Res. 2018. 10.1111/hepr.13306 30597744

[30] Wu D, Chen E, Liang T, Wang M, Chen B, Lang B, et al Predicting the risk of postoperative liver failure and overall survival using liver and spleen stiffness measurements in patients with hepatocellular carcinoma. Medicine. 2017;96(34):e7864 10.1097/MD.0000000000007864 28834899 PMC5572021

[31] Marasco G, Colecchia A, Dajti E, Ravaioli F, Cucchetti A, Cescon M, et al Prediction of posthepatectomy liver failure: Role of SSM and LSPS. J Surg Oncol. 2019;119(3):400–1. 10.1002/jso.25345 30561034

[32] Palmes D, Budny TB, Dietl KH, Herbst H, Stratmann U, Spiegel HU. Detrimental effect of sinusoidal overperfusion after liver resection and partial liver transplantation. Transpl Int. 2005;17(12):862–71. 10.1007/s00147-005-0809-9 15856174

[33] Cescon M, Vetrone G, Grazi GL, Ramacciato G, Ercolani G, Ravaioli M, et al Trends in perioperative outcome after hepatic resection: analysis of 1500 consecutive unselected cases over 20 years. Annals of surgery. 2009;249(6):995–1002. 10.1097/SLA.0b013e3181a63c74 19474679

[34] Hester CA, El Mokdad A, Mansour JC, Porembka MR, Yopp AC, Zeh HJ 3rd, et al Current Pattern of Use and Impact of Pringle Maneuver in Liver Resections in the United States. J Surg Res. 2019;239:253–60. 10.1016/j.jss.2019.01.043 30856518

[35] Andreatos N, Amini N, Gani F, Margonis GA, Sasaki K, Thompson VM, et al Albumin-Bilirubin Score: Predicting Short-Term Outcomes Including Bile Leak and Post-hepatectomy Liver Failure Following Hepatic Resection. Journal of gastrointestinal surgery: official journal of the Society for Surgery of the Alimentary Tract. 2017;21(2):238–48. 27619809 10.1007/s11605-016-3246-4

[36] Chen JS, Huang JQ, Chen XL, Zhan GF, Feng JT. Risk Factors Associated with Intraoperative Major Blood Loss during Resection of Hepatocellular Carcinoma. Hepatogastroenterology. 2015;62(140):790–3. 26902002

[37] Rossler F, Sapisochin G, Song G, Lin YH, Simpson MA, Hasegawa K, et al Defining Benchmarks for Major Liver Surgery: A multicenter Analysis of 5202 Living Liver Donors. Annals of surgery. 2016;264(3):492–500. 10.1097/SLA.0000000000001849 27433909

